# Ropeginterferon alfa-2b shows anti-polycythaemia vera activity without causing clinically significant anaemia

**DOI:** 10.1038/s44276-024-00076-4

**Published:** 2024-07-11

**Authors:** Keita Kirito, Albert Qin, Shanshan Suo, Rongfeng Fu, Daoxiang Wu, Toshiaki Sato, Oleh Zagrijtschuk, Kazuya Shimoda, Norio Komatsu, Jie Jin

**Affiliations:** 1 Department of Haematology and Oncology, University of Yamanashi, 1110 Shimokato, Chuo-shi, Yamanashi, 409-3898 Japan; 2 Medical Research & Clinical Operations, PharmaEssentia Corporation, Taipei, Taiwan; 3 The First Affiliated Hospital, Zhejiang University School of Medicine, Hangzhou, Zhejiang China; 4 State Key Laboratory of Experimental Haematology, National Clinical Research Centre for Blood Diseases, Institute of Haematology and Blood Diseases Hospital, Chinese Academy of Medical Sciences & Peking Union Medical College, Tianjin, 300020 China; 5 PharmaEssentia Biotech (Beijing) Limited, Beijing, China; 6 PharmaEssentia Japan K.K, Akasaka Centre Building 12F, 1‑3‑13 Moto‑akasaka, Minato-ku, Tokyo 107-0051 Japan; 7 PharmaEssentia USA, Burlington, MA USA; 8 Division of Haematology, Diabetes and Endocrinology, Department of Internal Medicine, Faculty of Medicine, University of Miyazaki, 5200 Kihara, Kiyotake‑cho, Miyazaki‑city, Miyazaki, 889‑1692 Japan; 9 Department of Haematology, Juntendo University School of Medicine, 2-1-1 Hongo, Bunkyo-ku, Tokyo 113-8421 Japan; 10 Department of Advanced Haematology, Juntendo University Graduate School of Medicine, 2-1-1 Hongo, Bunkyo-ku, Tokyo 113-8421 Japan

## Abstract

Anaemia could develop in polycythemia vera (PV) due to phlebotomy-caused iron-deficiency and cytotoxic effect of cytoreductive therapy. Ropeginterferon alfa-2b treatment was not associated with ≥grade 3 anaemia in two recent clinical studies of 78 patients with PV. Only four cases of grade 2 anaemias occurred, the anaemia resolved. The mean haemoglobin levels were above 120.0 g/L. Therefore, ropeginterferon alfa-2b treatment does not lead to clinically significant anaemia and appears to manage PV without affecting normal erythropoiesis. “Both trials were registered at clinicaltrials.gov (A19-201: NCT04182100; A20-202: NCT05485948)”.

Polycythaemia vera (PV) is a Philadelphia chromosome-negative myeloproliferative neoplasm (MPN) that, in most cases, harbour the Janus kinase 2 gene (*JAK2*) driver mutation *JAK2*V617F [1]. PV is characterised by an over-production of blood cells with increased haematocrit levels, which is a risk factor for thrombotic events (TEs) and cardiovascular mortality [1, 2]. Low-dose aspirin and phlebotomy are usually recommended for patients with low-risk PV (i.e., no history of thrombosis and age ≤60 years). The National Comprehensive Cancer Network (NCCN) recommends ropeginterferon alfa-2b (*BESREMi*®) as a preferred cytoreductive treatment for patients with low- or high-risk PV [3].

Ropeginterferon alfa-2b is a novel polyethylene glycol (PEG)-conjugated recombinant proline-interferon alpha (IFN-a) with a favourable *in vivo* pharmacokinetic (PK) profile [4, 5]. Ropeginterferon alfa-2b has demonstrated substantial anti-PV clinical activity, including complete haematologic response (CHR; defined as a haematocrit <45% without phlebotomy, a platelet count ≤ 400 × 10^9^/L, and a white blood cell count ≤10 × 10^9^/L) and a reduction in the *JAK2*V617F allele burden [6–9]. Ropeginterferon alfa-2b injection is approved for adult patients with PV at an initial dose of 100 µg (or 50 µg for patients already receiving cytoreductive therapy) with 50 µg incremental intrapatient increases in the dose up to a maximum recommended dose of 500 µg every two weeks. It can take several months to reach the plateau dose level [6]. An alternative dosing regimen with a higher starting dose of 250 µg and simpler intrapatient dose escalation to 500 µg every two weeks with flexible dose adjustment according to tolerability was explored as a treatment option. This regimen controlled PV effectively, as defined by the CHR, and was associated with a shorter time to achieve a CHR [8, 9]. In this report, we aimed to examine the data from the approved slow-dose titration and exploratory higher starting dose regimens focusing on the dynamics of haemoglobin (Hgb) and the occurrence of anaemia. Anaemia is important in the context of PV treatment for several reasons. First, patients who undergo frequent phlebotomy may suffer from symptomatic iron deficiency, leading to anaemia [10]. Anaemia and symptoms can negatively affect the patient well-being and should be avoided in patients with PV and MPNs. The symptoms include headache, insomnia, concentration difficulties, dizziness, restless legs and may coincide and potentiate the disease-related symptoms of the underlying MPN [11–13]. Commonly used agents in the PV treatment cause anaemia in substantial numbers of cases ranging from 18% with hydroxyurea (HU) [14] to 72% with ruxolitinib [11, 15]. Anaemia is symptomatic in many cases and may limit the treatment dose or lead to treatment interruption if uncontrolled or severe cases are present. Association between venous thromboembolism and iron-deficiency anaemia has also been shown [16]. Thus, having an agent that can effectively control the elevated haematocrit without excessively suppressing the normal erythropoiesis is a major therapeutic advantage.

An important question regarding ropeginterferon alfa-2b in this context is whether the control of haematocrit is commonly accompanied by clinically significant anaemia, i.e., at the ≥grade 3 level or at the moderate, grade 2 level, but the anaemia is persistent and unmanageable. We therefore performed a retrospective analysis of the effect of ropeginterferon alfa-2b on Hgb levels at various time points or on the occurrence of anaemia with the data available from our two prospective clinical studies in patients with PV.

A19-201 was conducted in Japan with the approved slow-dosing regimen, and A20-202 was conducted in China with a higher starting dose regimen, i.e., the 250–350–500 μg regimen. The study population and demographics of 78 patients in these two studies were reported previously [7, 9]. Patients were prospectively treated with ropeginterferon alfa-2b under the Institutional Review Board (IRB)-approved protocols in both studies. The mean Hgb levels at baseline were 137.3 g/L in A19-201 and 152.0 g/L in A20-202. All the observed cases of anaemia in both studies were mild or moderate. No grade 3 or 4 anaemia was observed. Only one patient in A19-201 and three patients in A20-202 developed grade 2 anaemia (Table 1). All patients with grade 2 anaemia quickly recovered within an average of 2.4 weeks after the dose of ropeginterferon alfa-2b was reduced, except for one patient whose grade 2 anaemia resolved without any dose reduction. Ropeginterferon alfa-2b treatment led to CHRs in both studies (71.4% in the China PV study and 51.7% at week 52 in the Japan PV study). The median time to reach the first CHR was ~5.6 months with the higher starting dose and faster intrapatient dose titrations in A20-202. The median time to reach the first CHR was ~12 months in A19-201 [7, 9]. In A20-202, the median biweekly dosage per patient was 462.8 μg, which was greater than the 362.1 μg in A19-201. Over the 52-week treatment period in both studies, the Hgb levels in all patients did not decrease below 8.0 g/dL or were at levels suggesting severe anaemia. The Hgb levels decreased over time, and the mean or median levels were lower than the baseline levels in both studies (Fig. 1a, b). However, the mean or median Hgb levels remained above 120.0 g/L for 52 weeks of treatment (Fig. 1a, b). Therefore, no clinically significant anaemia was observed with ropeginterferon alfa-2b treatment, and the mean or median Hgb levels remained above 120.0 g/L throughout both studies. The higher starting dosing regimen led to a shorter median time to reach CHR and had a only numerically higher, but not clinically significant, occurrence of mild or moderate anaemia.

**Table 1:** Treatment-emergent adverse events of anaemia in two phase II PV studies with a total of 78 patients.

PV Study	Anaemia					
	Grade 1	Grade 2	Grade 3	Grade 4	Grade 5	Total
	*n* (%)	*n* (%)	*n* (%)	*n* (%)	*n* (%)	*n* (%)
A19-201 study [7]	3 (10.3%)	1 (3.4%)^a^	0	0	0	4 (13.7%)
A20-202 study [9]	8 (16.3%)	3 (6.1%)	0	0	0	11 (22.4%)

Anaemia was graded according to the Common Terminology Criteria for Adverse Events version 5.0 (CTCAE v.5.0). Grade 1: haemoglobin (Hgb) lower limit of normal (LLN) to 10 g/dL; Grade 2: Hgb 8–10 g/dL; Grade 3: Hgb < 8 g/dL; Grade 4: life-threatening consequences, urgent intervention indicated; Grade 5: death.

^a^Grade 2 anaemia was noted during the safety follow-up after the discontinuation of treatment in one patient.

**Fig. 1:**
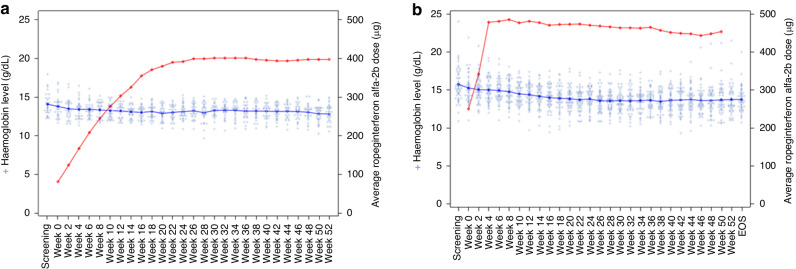
Haemoglobin (Hgb) levels in two clinical studies A19-201 and A20-202. **a** A19-201 study: Hgb level (blue line) vs. average ropeginterferon alfa-2b dose (red line). **b** A20-202 study: Hgb level (blue line) vs. average ropeginterferon alfa-2b dose (red line).

Severe or clinically significant anaemia can be a concerning clinical side effect in the treatment of patients with MPNs. This effect can be observed with the cytoreductive agent HU [17], as well as during treatment with ruxolitinib [15]. In our two clinical studies, we did not observe any ≥grade 3 anaemia, and the grade 2 or overall anaemia rates were numerically lower than previously reported for HU or ruxolitinib. Ropeginterferon alfa-2b injection is a new PEGylated IFN-α-based therapy that was approved for PV treatment by the European Commission in 2019, in the United States in 2021, and in Japan in 2023. Ropeginterferon alfa-2b induces substantial haematocrit control, leading to the independence from phlebotomy/erythrocyte apheresis to achieve a CHR. Ropeginterferon alfa-2b treatment at a fixed low dose level of 100 μg every 2 weeks was superior to phlebotomy alone in maintaining the haematocrit level without an anaemia issue. However, patients who switched to ropeginterferon alfa-2b treatment from phlebotomy alone might need higher doses to be effective [18]. Our analysis indicates that ropeginterferon alfa-2b treatment, either with the approved slow-dose titration regimen or with the dosing regimen of a higher starting dose and simpler intrapatient dose titrations, is not associated with severe or clinically significant anaemia with mean and median haemoglobin levels above the 120.0 g/L level. This finding is consistent with the fact that ropeginterferon alfa-2b is an IFN-α-based therapy and that type I IFNs can selectively suppress cell cycle progression accompanied by senescence entry and loss of tumorigenicity in neoplastic cells while leaving normal cells growing at the same condition largely unaffected [19]. Ropeginterferon alfa-2b treatment at two dosing regimens can achieve a CHR without causing clinically significant anaemia. Therefore, ropeginterferon alfa-2b treatment may have clinical anti-PV effects without affecting normal erythropoiesis. The finding may further support its use at a higher starting dose as an option to achieve a CHR rapidly without causing anaemia for patients with PV.

## Data Availability

The datasets generated during and/or analysed during the current study are available from the corresponding author on reasonable request.
